# Direct chemical editing of Gram‐positive bacterial cell walls via an enzyme‐catalyzed oxidative coupling reaction

**DOI:** 10.1002/EXP.20220010

**Published:** 2022-05-28

**Authors:** Hao‐Ran Jia, Ya‐Xuan Zhu, Yi Liu, Yuxin Guo, Sayed Mir Sayed, Xiao‐Yu Zhu, Xiaotong Cheng, Fu‐Gen Wu

**Affiliations:** ^1^ State Key Laboratory of Bioelectronics, School of Biological Science and Medical Engineering Southeast University Nanjing P. R. China

**Keywords:** biosensing, cell surface engineering, live cells, oxidative coupling, tyrosinase

## Abstract

Chemically manipulating bacterial surface structures, a cutting‐edge research direction in the biomedical field, predominantly relies on metabolic labeling by now. However, this method may involve daunting precursor synthesis and only labels nascent surface structures. Here, we report a facile and rapid modification strategy based on a tyrosinase‐catalyzed oxidative coupling reaction (TyOCR) for bacterial surface engineering. This strategy employs phenol‐tagged small molecules and tyrosinase to initiate direct chemical modification of Gram‐positive bacterial cell walls with high labeling efficiency, while Gram‐negative bacteria are inert to this modification due to the hindrance of an outer membrane. By using the biotin‒avidin system, we further present the selective deposition of various materials, including photosensitizer, magnetic nanoparticle, and horseradish peroxidase, on Gram‐positive bacterial surfaces, and realize the purification/isolation/enrichment and naked‐eye detection of bacterial strains. This work demonstrates that TyOCR is a promising strategy for engineering live bacterial cells.

## Graphical abstract

This work describes a mild and efficient enzyme‐catalyzed chemical reaction for the selective modification of Gram‐positive bacterial cell walls. The as‐designed technique can serve as a universal platform to functionalize the surfaces of live bacterial cells with diverse responsive materials, including small‐molecule fluorescent dyes, horseradish peroxidase, and magnetic nanoparticles.

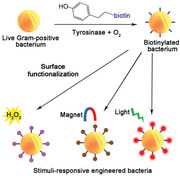

## 1 INTRODUCTION

Bacterial surface engineering, a set of biological techniques intended to manipulate the surface structures/components of bacterial cells, has found applications in a great diversity of settings, ranging from biosensing and biocatalysis to cancer therapeutics and vaccinology.^[^ ^1^ ^]^ Genetic cell surface display is among the earliest and well‐established techniques in this field. It works by fusing exogenous proteins or peptides with the carrier proteins located on the bacterial envelope at the genetic level so that the cell surface can express rationally designed recombinant proteins.^[^ ^2^ ^]^ However, genetic manipulation is technically demanding and falls short of the ability to engineer other nonproteinous biomolecules (e.g., sugars and lipids) or to functionalize bacteria with synthetic materials.^[^ ^1a^ ^]^ For these reasons, researchers have endeavored to develop a series of alternative approaches based on physical modification, including electrostatic adsorption,^[^ ^3^ ^]^ lipid encapsulation,^[^ ^4^ ^]^ and hydrophobic anchoring.^[^ ^5^ ^]^ Designed as more facile and maneuverable tools, these strategies are capable of depositing different functional materials on bacterial cell surfaces, albeit in a less controllable manner. The ease of operation has guaranteed their broad applications, but the rapid disassociation of the attached materials from the cell surface, a major drawback encountered by most physical modification strategies,^[^ ^6^ ^]^ greatly precludes them from being applied in cases where long‐lasting cell surface decoration is needed.

In recent years, chemical surface display in bacteria emerges as a superior method that combines the good controllability of its genetic counterpart and the general applicability of physical modification.^[^ ^1a^ ^]^ One class of well‐established techniques for this purpose is metabolic labeling, through which unnatural metabolic precursors bearing fluorophores or chemical handles (e.g., bioorthogonal groups) can be utilized by the cell's intrinsic biosynthetic machinery so that nascent biomolecules of interest are covalently functionalized.^[^ ^7^ ^]^ Such strategies enable precise manipulation of not only proteins but also glycolipids^[^ ^8^ ^]^ and peptidoglycans^[^ ^9^ ^]^ in bacterial envelopes. Another promising class of labeling techniques aims to conjugate exogenous decorative materials directly with some native reactive groups (e.g., amine and thiol) of cell surface‐located biomolecules, and allows convenient, rapid, and universal modification of live cell surfaces regardless of the metabolic activity of the cell. Achieving this purpose calls for suitable chemical reactions that should be compatible with the physiological environment, mild to cell survival, and as fast as possible, but currently available candidates remain very rare with only a few examples.^[^ ^10^ ^]^ Among them, the most widely used reaction is probably the one between *N*‐hydroxysuccinimide (NHS) ester and a primary amine, which, however, still suffers from limitations such as undesired hydrolysis of NHS ester.^[^ ^7^ ^]^ Moreover, most of the established techniques are designed for mammalian cells rather than bacteria. As a consequence, developing direct bioconjugation approaches for bacterial surface modification is of tremendous practical and scientific values.

**SCHEME 1:**
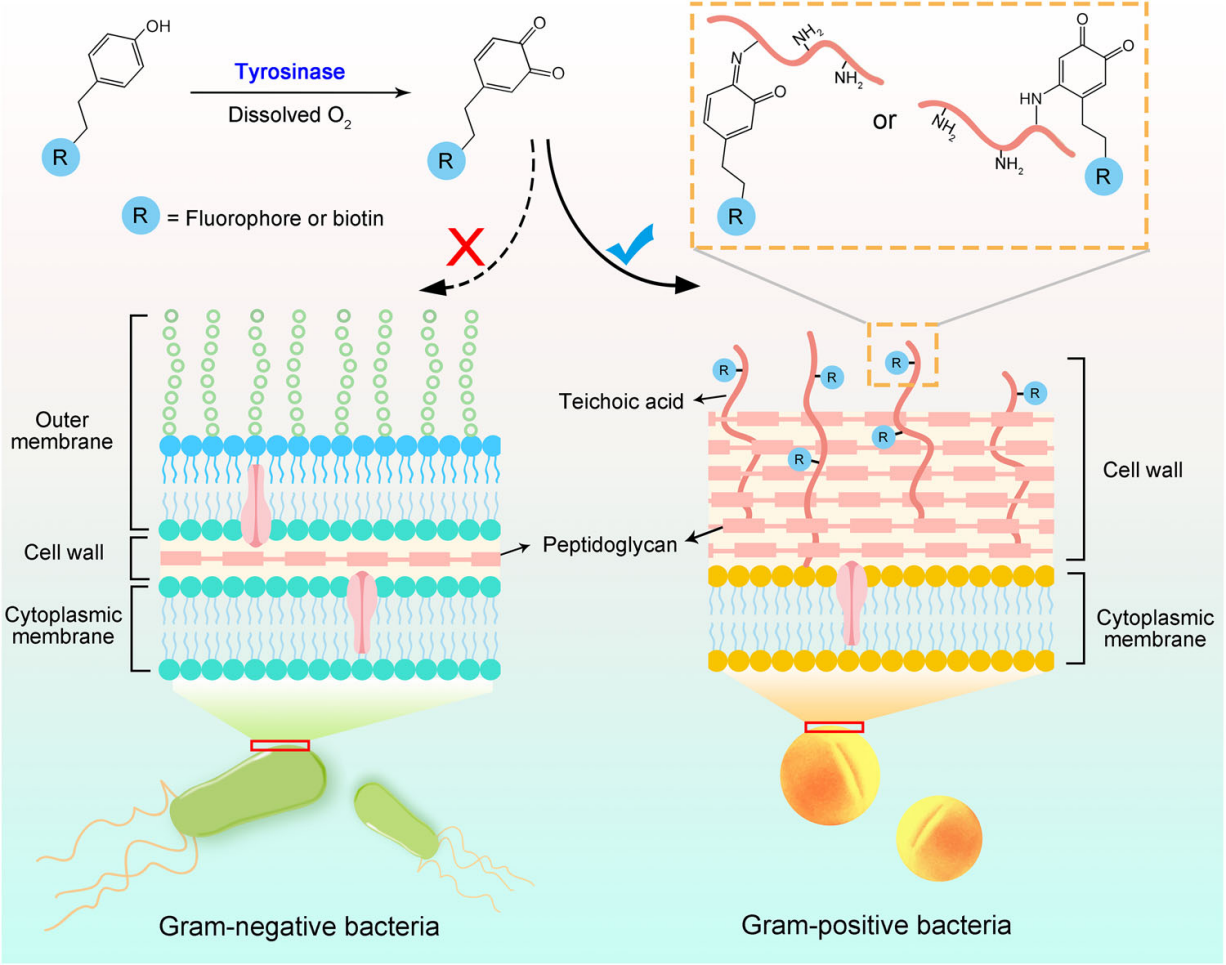
Reaction mechanism of the bioconjugation between phenol‐tagged molecules and teichoic acids embedded in the Gram‐positive bacterial cell wall

In cell biology, tyrosinase is an endogenous enzyme in melanosomes able to catalyze the oxidation of tyrosine into dopaquinone, which then undergoes intramolecular cyclization and polymerization to form melanin. The basic principle of this biological process lies in tyrosinase's capability to convert phenols or catechols into *o*‐quinones using dissolved oxygen as the sole oxidant under mild ambient conditions. In recent years, researchers have exploited the advantageous properties of tyrosinase and, considering the high reactivity of *o*‐quinones, have extended tyrosinase‐mediated oxidative coupling reactions (TyOCRs) to materials science, particularly the modification of polymers,^[^ ^11^ ^]^ proteins,^[^ ^12^ ^]^ and nanoparticles.^[^ ^13^ ^]^ For example, Maza and co‐workers designed an efficient coupling method based on abTYR, a commercially available tyrosinase extracted from *Agaricus bisporus* (a button mushroom), for the covalent linkage of phenol/catechol precursors with N‐terminal proline residues in proteins.^[^ ^12b^ ^]^ Herein, we present for the first time that TyOCRs can be applied to the chemical modification of live bacterial cells. In our design, chemical reporters (i.e., fluorophores and biotin) tagged with phenol groups can be conjugated to teichoic acids (TAs), a major component of Gram‐positive bacterial cell walls, in the presence of tyrosinase with high modification efficiencies (Scheme 1). Interestingly, such a strategy is not applicable to Gram‐negative bacteria, which typically lack TAs in their cell wall structures and are covered by less permeable outer membranes. Benefiting from the excellent specificity, we display a series of technical examples of functionalizing Gram‐positive bacteria for fluorescence imaging, photodynamic inactivation, magnetic cell separation, and naked‐eye biosensing. This tyrosinase‐catalyzed bacterial modification technique is anticipated to enrich the toolbox for cell surface engineering and find promising applications in various biomedical fields.

## 2 RESULTS AND DISCUSSION

### 2.1 Selective fluorescence labeling of Gram‐positive bacteria

**FIGURE 1:**
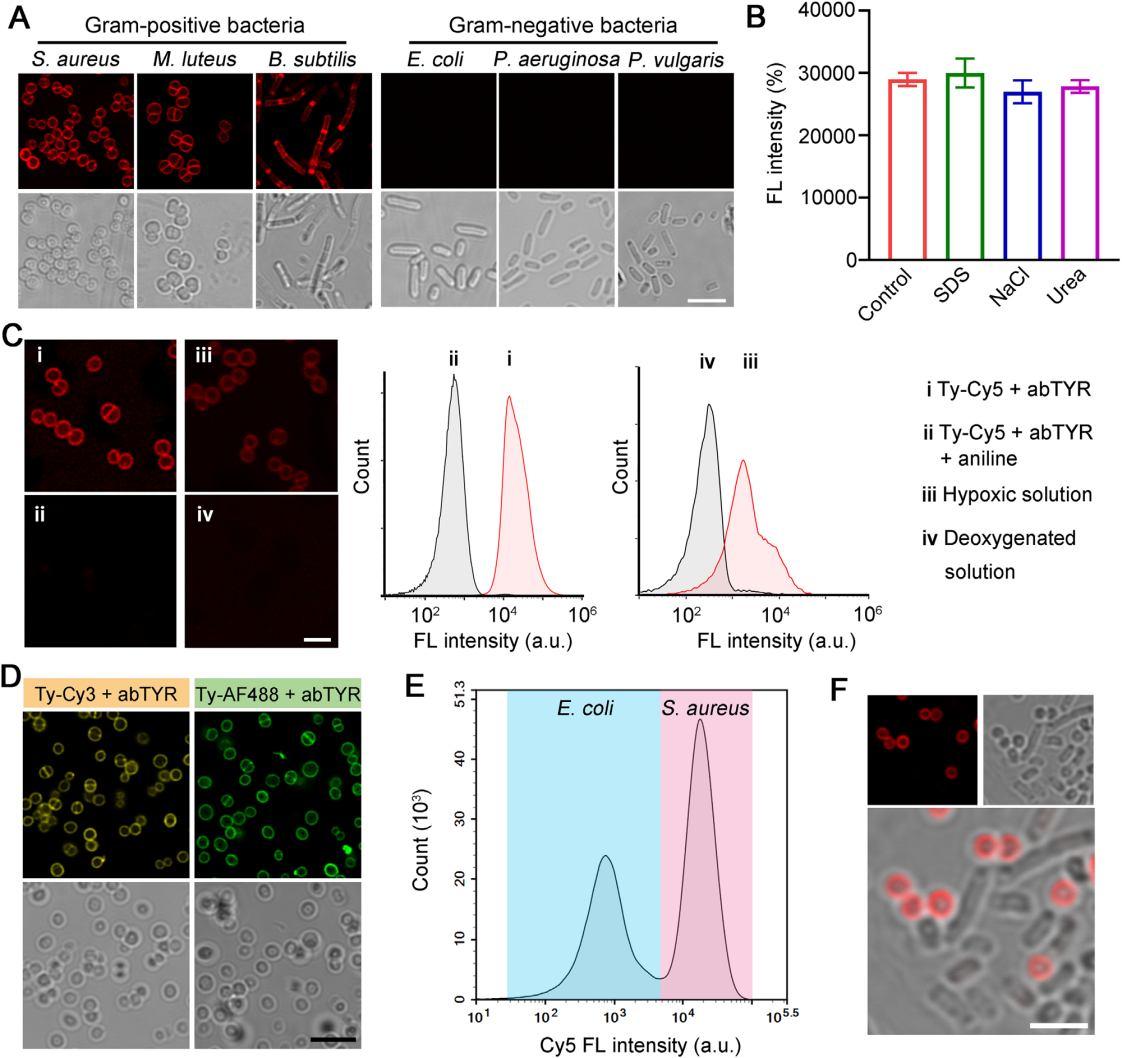
Selective fluorescence labeling of Gram‐positive bacterial cell walls. (A) Confocal images of different types of bacterial cells treated with abTYR (0.17 µM) and Ty‐Cy5 (1 µg mL^−1^) for 10 min. Before imaging, the bacteria were washed with phosphate‐buffered saline (PBS) for three times. Scale bar = 5 µm. (B) Fluorescence (FL) intensities of Cy5‐stained *S. aureus* bacterial cells (the bacterial number in each sample was the same) after being treated with PBS (control), 0.1% SDS, 0.5 M NaCl, and 50 mM urea solutions, respectively, as measured by flow cytometry. (C) Confocal images and flow cytometric results of *S*. *aureus* bacteria after different treatments as indicated. Aniline was used as a competitor to consume *o*‐quinone‐Cy5. The participation of oxygen in TyOCR was demonstrated by bacterial labeling in hypoxic and deoxygenated solutions. Scale bar = 2.5 µm. (D) Confocal images of *S*. *aureus* bacteria that were stained by Ty‐Cy3 (1 µg mL^−1^) or Ty‐AF488 (1 µg mL^−1^) in the presence of abTYR (0.17 µM). Before imaging, the bacteria were washed with PBS for three times. Scale bar = 5 µm. Flow cytometric result (E) and confocal fluorescence images (F) of a mixed suspension of *E*. *coli* and *S*. *aureus*, which was treated with Ty‐Cy5 (1 µg mL^−1^) and abTYR (0.17 µM) at room temperature for 10 min. Before measurements, the mixed suspension was washed with PBS for three times. Scale bar = 2.5 µm

We began by employing different bacteria as experimental models to explore whether abTYR could mediate the conjugation of phenol‐tagged molecules to bacterial cell surfaces. Initially, *Staphylococcus aureus* (*S*. *aureus*), *Micrococcus luteus* (*M*. *luteus*), and *Bacillus subtilis* (*B*. *subtilis*), three Gram‐positive bacterial species, were cultured to reach the exponential growth phase. These cells were then treated with abTYR (0.17 µM) and cyanine 5 tyramide (Ty‐Cy5, 1 µg mL^−1^) at room temperature for 10 min, followed by confocal fluorescence imaging. We strikingly observed that all the bacterial surfaces were labeled by red fluorescence (Figure 1A). To rule out the possibility of physical adsorption, we treated the stained *S*. *aureus* bacterial cells with NaCl (to dissociate electrostatic interaction), sodium dodecyl sulfate (SDS, to interrupt formed hydrophobic interaction), or urea (to form competitive hydrogen bonds), neither of which could weaken the introduced fluorescence signals (Figure 1B), suggesting that the labeling of Cy5 was covalent. Furthermore, we challenged the staining system by adding excessive aniline, which can consume *o*‐quinones and thus compete with possible reaction sites on the bacterial surface, or by depleting dissolved molecular oxygen to block the oxidation of phenols into *o*‐quinones. Confocal microscopic and flow cytometric data both demonstrated that these treatments almost entirely suppressed the ability of Ty‐Cy5 to label *S*. *aureus* bacteria (Figure 1C). Aside from Ty‐Cy5, we proved that other phenol‐tagged fluorophores such as cyanine 3 tyramide (Ty‐Cy3) and Alexa Fluor 488 tyramide (Ty‐AF488) could also realize similar imaging performance (Figure 1D), implying the generality of the TyOCR‐based bacterial staining method. Next, we sought to investigate whether this strategy is applicable to Gram‐negative bacteria. Interestingly, no evident fluorescence signal could be detected in the three Gram‐negative bacterial cells *Escherichia coli* (*E*. *coli*), *Pseudomonas aeruginosa* (*P*. *aeruginosa*), and *Proteus vulgaris* (*P*. *vulgaris*) after receiving an identical staining treatment (Figure 1A). We further confirmed that when *E*. *coli* coexisted with *S*. *aureus*, only the latter could be effectively labeled by Ty‐Cy5 (Figure 1E,F). To be noted, such specificity is exclusive to viable cells, because dead bacteria with compromised surface integrity allowed fluorescent probes to arbitrarily enter the cells (Figure S1). Together, these data suggest that TyOCRs can realize selective cell surface labeling of live Gram‐positive bacteria.

### 2.2 Teichoic acids are the labeling sites

**FIGURE 2:**
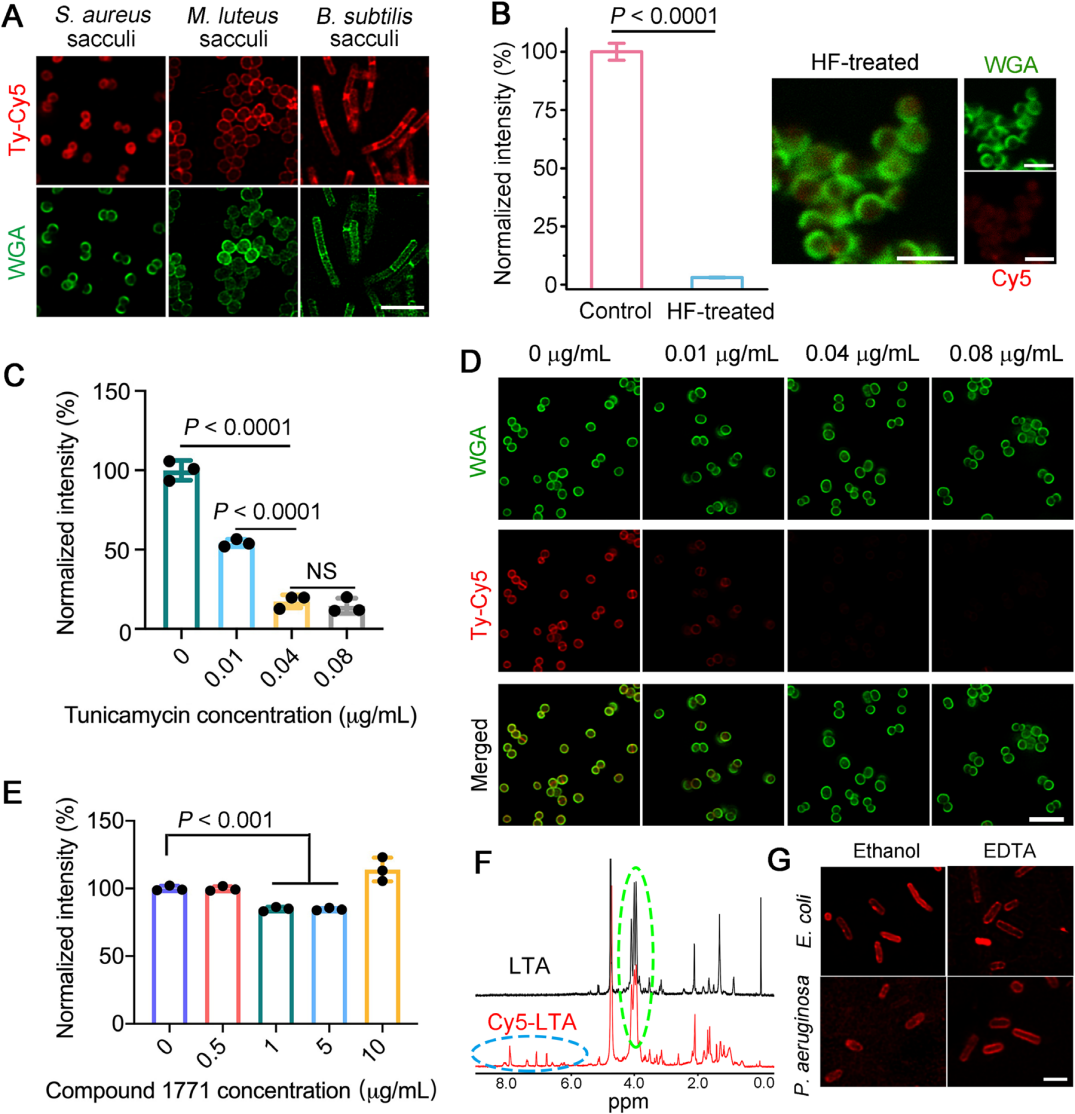
WTA is the major labeling site of TyOCR. (A) Confocal fluorescence images of different sacculi purified from their corresponding live bacterial cells labeled by Ty‐Cy5. Before imaging, the sacculi were stained by Alexa Fluor 488‐labeled wheat germ agglutinin (WGA‐AF488, 5 µg mL^−1^) to indicate cell wall structures. Scale bar = 5 µm. (B) Normalized fluorescence intensities and confocal fluorescence images of Ty‐Cy5‐labeled *S. aureus* sacculi with or without (control) the HF treatment. Before imaging, the sacculi were stained by WGA‐AF488 (5 µg mL^−1^). Scale bars = 2 µm. Statistical data were analyzed by unpaired Student's *t*‐test. Flow cytometric results (C) and confocal fluorescence images (D) showing the efficiencies of TyOCR‐based fluorescence labeling in *S*. *aureus* bacteria that were pretreated with different concentrations of tunicamycin. Statistical significance between the indicated groups was calculated using one‐way analysis of variance (ANOVA, "NS" stands for nonsignificant difference). Before imaging, the bacteria were first stained by Ty‐Cy5 (1 µg mL^−1^) in the presence of abTYR (0.17 µM) and then stained by WGA‐AF488 (5 µg mL^−1^), followed by PBS washing for three times. (E) Flow cytometric results of *S*. *aureus* bacteria that were pretreated with different concentrations of compound 1771 and then subjected to TyOCR labeling. Statistical significance between the indicated groups was calculated using one‐way ANOVA. (F) ^1^H NMR spectra of LTA and Cy5‐LTA. The green and blue dashed circles indicate the characteristic peaks of LTA and Cy5, respectively. (G) Confocal fluorescence images of ethanol/EDTA‐pretreated *E*. *coli* and *P*. *aeruginosa* bacteria after being stained by Ty‐Cy5 via the TyOCR‐based labeling method. Scale bar = 2.5 µm

The cell surface of Gram‐positive bacteria typically consists of an outermost protective shell called the cell wall and an underlying cytoplasmic membrane. To determine the major labeling site of this modification technique, we converted the above Ty‐Cy5‐labeled Gram‐positive bacteria into bacterial exoskeletons (sacculi) that only retain the basic structure of the cell wall without cytoplasmic membrane or intracellular content. Confocal imaging data revealed that the red fluorescence signals were still maintained in these sacculi (Figure 2A), indicating the critical role of the cell wall in the fluorescence labeling. In general, the Gram‐positive bacterial cell wall is primarily composed of TA and peptidoglycan, wherein TA possesses abundant free primary amines in its alanine residues,^[^ ^14^ ^]^ and thus has been recognized as an effective reaction site for bioconjugate chemistry.^[^ ^15^ ^]^ We deduced that TA might participate in the TyOCR‐based fluorescence labeling because *o*‐quinone can readily react with the amine group.^[^ ^16^ ^]^ To confirm this, we employed hydrofluoric acid (HF) to treat the above‐prepared *S*. *aureus* sacculi, a well‐established method to remove TA from the cell wall structure of Gram‐positive bacteria by cleaving phosphodiester bonds.^[^ ^17^ ^]^ As expected, confocal images and quantitative data revealed that the fluorescence signals in the HF‐treated sacculi almost disappeared (Figure 2B), suggesting that Cy5 was dominantly linked to TA.

The cell wall of Gram‐positive bacteria comprises two types of TA, that is, wall TA (WTA) and lipoteichoic acid (LTA), which are covalently attached to peptidoglycan and cytoplasmic membrane, respectively. We excitingly found that after *S*. *aureus* bacteria were treated with increasing concentrations of tunicamycin, an inhibitor of WTA biosynthesis,^[^ ^18^ ^]^ the TyOCR‐based modification strategy gradually lost its capability to label these WTA‐deficient bacteria (Figure 2C,D). The elimination of WTA (using 0.08 µg mL^−1^ tunicamycin) from *S*. *aureus* cell wall accounted for an approximately 85% decrease in Cy5 signal. Likewise, we selectively suppressed the synthesis of LTA in *S*. *aureus* using a previously reported inhibitor, compound 1771,^[^ ^19^ ^]^ and discovered that the level of Cy5 fluorophores conjugated to these treated bacteria declined by approximately 15% (at 1 and 5 µg mL^−1^) compared with that in the control group (Figure 2E). The abnormal signal increase in the 10 µg mL^−1^ group was probably ascribed to the presence of dead bacteria that were killed by compound 1771. These data offer strong evidence that both WTA and LTA participate in the TyOCR‐based modification and WTA is the primary labeling site. To further corroborate the conclusion, commercial LTA purified from *S*. *aureus* was employed for the mechanistic study, and its amine content was quantified to be 2.0 × 10^‒4^ mol g^−1^ via the ninhydrin test. The successful conjugation of Ty‐Cy5 with the LTA polymer via TyOCR was verified by ultraviolet‒visible (UV‒vis) spectroscopy and ^1^H nuclear magnetic resonance (NMR) spectroscopy (Figure S2 and Figure 2F).

We then attempted to explain why the TyOCR‐based modification strategy failed to effectively label Gram‐negative bacteria under our tested conditions. On the one hand, the Gram‐negative bacterial cell wall lacks TA and thus cannot provide as many reaction sites for *o*‐quinone‐Cy5 (the oxidized form of Ty‐Cy5) as the Gram‐positive cell wall. On the other hand, we deduced that the outer membrane, an asymmetric lipid bilayer surrounding the Gram‐negative bacterial cell wall, may play a critical role because this barrier has been reported to reduce the efficacy of some antibacterial materials.^[^ ^20^ ^]^ To validate this hypothesis, we treated *E*. *coli* and *P*. *aeruginosa* bacteria with ethanol or ethylenediaminetetraacetic acid (EDTA) disodium salt, both of which can induce the disassociation of lipopolysaccharide (LPS, a major component of the bacterial outer membrane). Confocal imaging demonstrated that these ethanol/EDTA‐pretreated bacteria were unable to resist Ty‐Cy5 labeling, as evidenced by the red fluorescence on their cell surface (Figure 2G), which clearly indicated the crucial role of LPS in paralyzing the TyOCR‐based modification strategy. Collectively, the above results unravel that phenol‐tagged fluorophores can covalently modify the TA (primarily WTA) of Gram‐positive bacterial cell wall in the presence of abTYR and molecular oxygen; however, Gram‐negative bacteria are recalcitrant to this reaction due to the hindrance of LPS.

**FIGURE 3:**
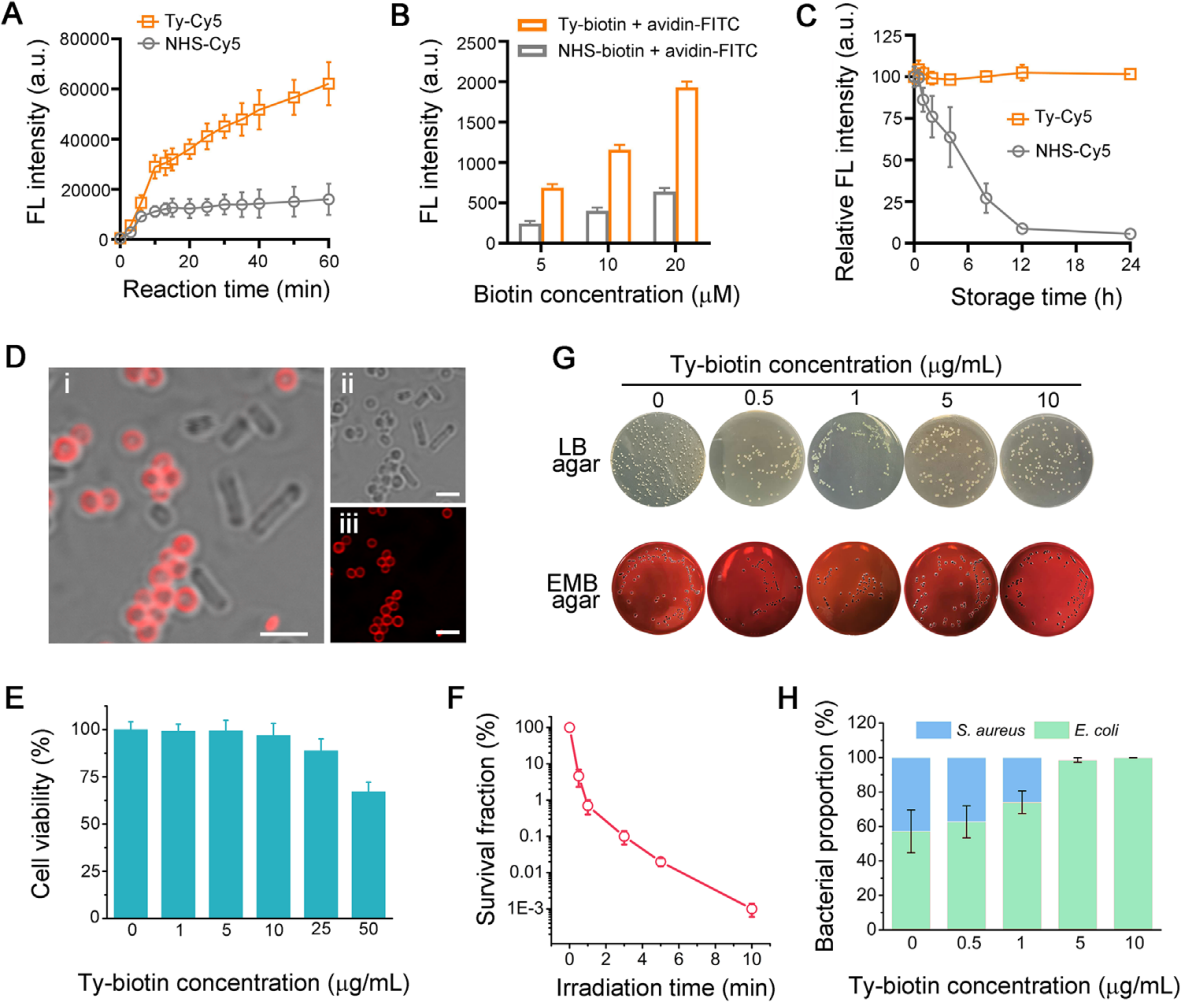
Comparison of different labeling strategies and photodynamic inactivation evaluations. (A) Fluorescence (FL) intensities of *S. aureus* bacteria that were reacted with Ty‐Cy5 (2 µM, in the presence of abTYR) or NHS‐Cy5 (2 µM) for different time periods, as measured by flow cytometry. (B) FL intensities of *S*. *aureus* bacteria that were first treated with different concentrations of Ty‐biotin (in the presence of abTYR) or NHS‐biotin for 20 min, washed with PBS, and further incubated with fluorescein isothiocyanate (FITC)‐labeled avidin (avidin‐FITC, 5 µg mL^−1^), as measured by flow cytometry. (C) Relative FL intensities of *S*. *aureus* bacteria stained by Ty‐Cy5 (2 µM, in the presence of abTYR) or NHS‐Cy5 (2 µM) for 20 min. Before staining, Ty‐Cy5 and NHS‐Cy5 were freshly dissolved in PBS (pH = 7.4) and stored at room temperature for different time periods as indicated. (D) Confocal images of a mixture of *E*. *coli* and *S*. *aureus* bacteria that were first treated with Ty‐biotin and abTYR and then incubated with avidin‐Ce6 (Ce6: 5 µg mL^−1^). Before imaging, the bacteria were washed with PBS three times. Panels (i)‒(iii) indicate merged, bright field, and fluorescence images, respectively. Scale bars = 2.5 µm. (E) Relative cell viabilities of *S*. *aureus* bacteria modified by different concentrations of Ty‐biotin via the abTYR‐mediated modification strategy. (F) Survival fractions of avidin‐Ce6‐modified *S*. *aureus* bacteria after being irradiated by white light (5 mW cm^−2^) for different time periods. (G) Representative photographs of LB and EMB agar plates after the photodynamic inactivation assays against *S*. *aureus*/*E*. *coli* mixtures, which were first pretreated with Ty‐biotin (0‒10 µg mL^−1^) and abTYR (0.17 µM), incubated with avidin‐Ce6 (Ce6: 5 µg mL^−1^), and irradiated by white light (5 mW cm^−2^) for 3 min. (H) Quantitative results showing the proportions of *S*. *aureus* and *E*. *coli* bacteria after different treatments as described in (G).

For cell surface modification, the most common bioconjugate method that can directly label native cell surface components is arguably the NHS ester chemistry.^[^ ^7^ ^]^ Given that this method also exploits amines as reactants, we confirmed its ability to covalently modify the cell surfaces of different Gram‐positive bacteria by using NHS ester‐functionalized cyanine 5 (NHS‐Cy5) as the labeling reagent (Figure S3a). Interestingly, in contrast to TyOCR, NHS‐Cy5 easily stained *E*. *coli* bacteria (Figure S3b,c), suggesting the conjugation of Cy5 to their outer membrane proteins. We infer that the reason for this difference probably lies in the inaccessibility of outer membrane proteins to TyOCR, because they have been reported to tightly interact with LPS.^[^ ^21^ ^]^ Of note, in terms of Gram‐positive bacteria, the labeling efficiency and modification density of NHS esters were found to be significantly lower than those of phenol‐tagged molecules (Figure 3A,B). Additionally, NHS ester is prone to hydrolysis in aqueous solution, as evidenced by the observation that NHS‐Cy5 gradually lost the capability to label *S*. *aureus* bacteria in PBS within 12 h (Figure 3C), which demands that the working solutions of NHS ester‐functionalized probes should be freshly prepared and used immediately. Taken together, the above results indicate that TyOCR can act as a superior strategy over NHS ester reaction for Gram‐positive bacterial cell surface modification.

### 2.3 Photodynamic inactivation against Gram‐positive bacteria

Next, we attempted to generalize this labeling strategy to establish a versatile platform for bacterial cell surface engineering by employing the biotin‒avidin system. To this end, biotinyl tyramide (Ty‐biotin) and abTYR were used to selectively engineer Gram‐positive bacterial cell walls in the presence of Gram‐negative bacteria, and then the biotinylated bacteria were further treated with Ce6 (a Type II photosensitizer)‐conjugated avidin. Confocal images in Figure 3D revealed that avidin‐Ce6 selectively bound to the surfaces of *S*. *aureus* rather than those of *E*. *coli*, although both of the bacteria previously received the TyOCR‐based biotinylation. In this scenario, only *S*. *aureus* bacteria could be killed by Ce6 under light exposure. The optimal concentration of Ty‐biotin for chemical modification was determined to be 10 µg mL^−1^ because higher doses displayed a toxic effect on the bacterial cells (Figure 3E). Antibacterial assays demonstrated that exposing the Ce6‐labeled *S*. *aureus* bacteria to mild (5 mW cm^−2^) white light irradiation for 10 min was sufficient to realize an approximately 99.999% bactericidal efficiency (Figure 3F). To further test the bacterial killing specificity of the as‐designed antibacterial system, we assessed its bactericidal efficiency against a mixture of *S*. *aureus* and *E*. *coli*. Total colony forming units (CFUs) and *E*. *coli* CFUs were counted using lysogeny broth (LB) agar and eosin methylene blue (EMB) agar plating methods, respectively (Figure 3G). Quantitative results displayed that *E*. *coli* gradually gained a preponderance from the original 57% to almost 100% with the increase of Ty‐biotin concentration (Figure 3H), implying the efficient elimination of *S*. *aureus* from the mixed system. Such a selective Gram‐positive bacterial killing effect may allow us to purify/isolate/enrich Gram‐negative bacterial strains in laboratory settings.

### 2.4 Magnetic isolation of Gram‐positive bacteria

**FIGURE 4:**
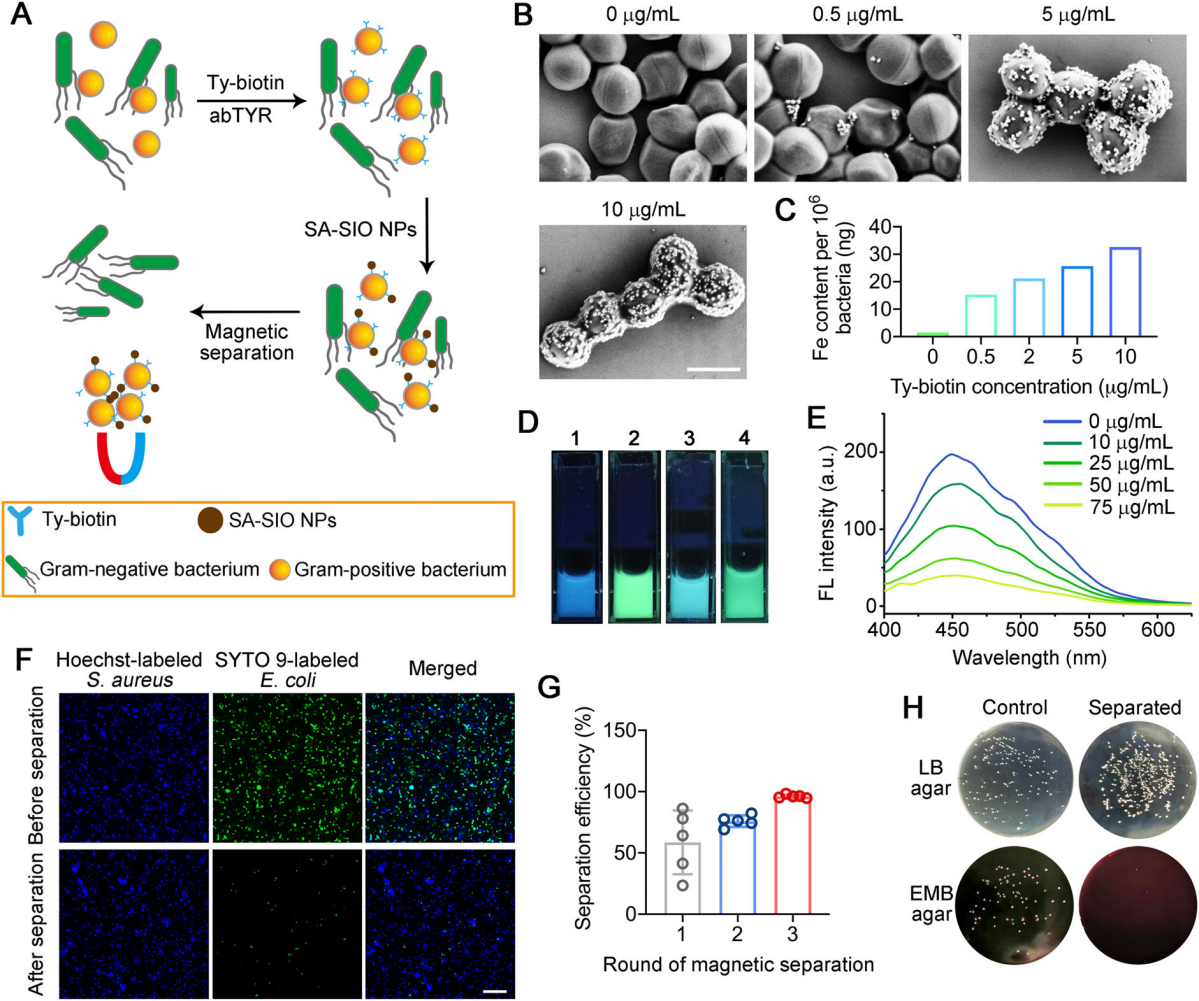
Magnetic isolation of Gram‐positive bacteria. (A) Schematic diagram illustrating the magnetic separation of Gram‐positive bacteria from Gram‐negative bacteria based on the TyOCR‐based modification strategy. (B) SEM images of *S. aureus* bacteria that were first treated with different concentrations of Ty‐biotin and abTYR (0.17 µM) and then labeled by SA‐SIO NPs (50 µg mL^−1^). Scale bar = 1 µm. (C) ICP‐MS results showing the Fe contents in SA‐SIO NP‐modified *S*. *aureus* bacteria that were prepared as described in (B). (D) Photographs of different bacterial suspensions under a UV lamp. To be specific, **1** is a Hoechst 33342‐stained *S*. *aureus* suspension; **2** is an SYTO 9‐stained *E*. *coli* suspension; **3** is a mixture of an equal volume of **1** and **2**; **4** is the resultant suspension of **3** that was first treated with Ty‐biotin (10 µg mL^−1^) plus abTYR (0.17 µM), then incubated with 50 µg mL^−1^ SA‐SIO NPs, and finally subjected to magnetic separation. (E) Fluorescence emission spectra of Hoechst 33342 in **3** that was first treated with Ty‐biotin (10 µg mL^−1^) plus abTYR (0.17 µM), then labeled by different concentrations of SA‐SIO NPs (0−75 µg mL^−1^), and finally subjected to magnetic separation. (F) Confocal fluorescence images of **3** before and after the magnetic separation treatment. Scale bar = 25 µm. (G) Efficiencies of isolating *S*. *aureus* from a mixture of *S*. *aureus* and *E*. *coli* after different rounds of magnetic separation. (H) Representative photographs of LB and EMB plates spread with mixed *S*. *aureus*/*E*. *coli* suspensions that were pretreated with or without (control) three rounds of magnetic separation

Aside from introducing small‐molecule tags to bacterial surfaces, we further explored the possibility of modifying cell walls with functional nanomaterials. To this end, streptavidin (SA)‐coated superparamagnetic iron oxide NPs (SA‐SIO NPs) were chosen as a model material and incubated with the bacteria that were previously biotinylated via the TyOCR‐based modification strategy. We anticipate that this design may be suitable for isolating Gram‐positive bacteria from Gram‐negative bacteria under an external magnetic field (Figure 4A). Scanning electron microscopy (SEM) images revealed that SA‐SIO NPs were successfully modified on the bacterial surfaces, and more importantly, the labeling densities of SA‐SIO NPs could be finely tuned by varying the concentration of Ty‐biotin applied in the first step (Figure 4B). Such result was in good accordance with the trend displayed by the quantitative data (Figure 4C), as measured by inductively coupled plasma mass spectrometry (ICP‐MS). In contrast, no SA‐SIO NP was observed in the *E*. *coli* samples subjected to the same two‐step modification (Figure S4). These results encouraged us to further test whether this strategy is feasible for bacterial isolation.

To begin with, we separately labeled *E*. *coli* and *S*. *aureus* with SYTO 9 (a green‐fluorescent nucleic acid stain) and Hoechst 33342 (a blue‐fluorescent nucleic acid stain) so that their suspensions could be easily discerned under a UV lamp (Figure 4D). An equal volume mixture of the two bacterial suspensions was characterized by cyan fluorescence. Next, the mixture was subjected to the two‐step surface coating of SA‐SIO NPs and placed under a strong magnetic field. We strikingly observed that the mixed suspension was restored to green fluorescence under the excitation of UV light (Figure 4D), which suggested the removal of blue‐fluorescent *S*. *aureus* bacteria from this mixed system in response to the magnetic field. We also confirmed this observation by measuring the fluorescence spectra of a series of magnetically separated suspensions. The results presented a decreasing trend in the fluorescence intensities of Hoechst 33342, which labeled the *S*. *aureus* bacteria, as we gradually increased the concentration of SA‐SIO NPs used for magnetic separation (Figure 4E). Therefore, the above data indicate that the abTYR‐mediated modification strategy can be tailored for magnetic isolation of Gram‐positive bacteria.

We then focused on the isolation efficiency of the as‐designed system. Magnetically enriched bacteria (i.e., those retained in separation columns) from the abovementioned experiments were collected for fluorescence imaging. Confocal images displayed that the majority of the bacteria were Hoechst 33342‐labeled *S*. *aureus*, suggesting a high separation efficiency, albeit still concomitant with a small proportion of SYTO 9‐labeled *E*. *coli* (Figure 4F). We consider that the remaining *E*. *coli* bacteria are not magnetically responsive but, based on a reasonable scenario, were nonspecifically encapsulated by an overwhelming number of SA‐SIO NP‐coated *S*. *aureus* bacteria, as driven by the strong magnetic force. As expected, we indeed observed large *S*. *aureus* aggregates where a few *E*. *coli* bacteria were sparsely embedded (Figure S5). To solve this issue, a feasible solution is to increase the rounds of magnetic separation, and before each separation, the sample should be vigorously pipetted to redisperse the bacteria. Quantitative data shown in Figure 4G,H proved that these procedures significantly improved the purity of isolated *S*. *aureus* from 58% after one round of separation to over 96% after three rounds. In addition, the cell viabilities of these magnetically isolated bacteria were not affected compared with untreated bacteria (Figure S6). Together, the above results demonstrate that the TyOCR‐based modification strategy can serve as a facile, rapid, and effective platform for the separation of Gram‐positive bacteria from a mixture of Gram‐positive and Gram‐negative bacteria.

### 2.5 Naked‐eye detection of Gram‐positive bacteria

**FIGURE 5:**
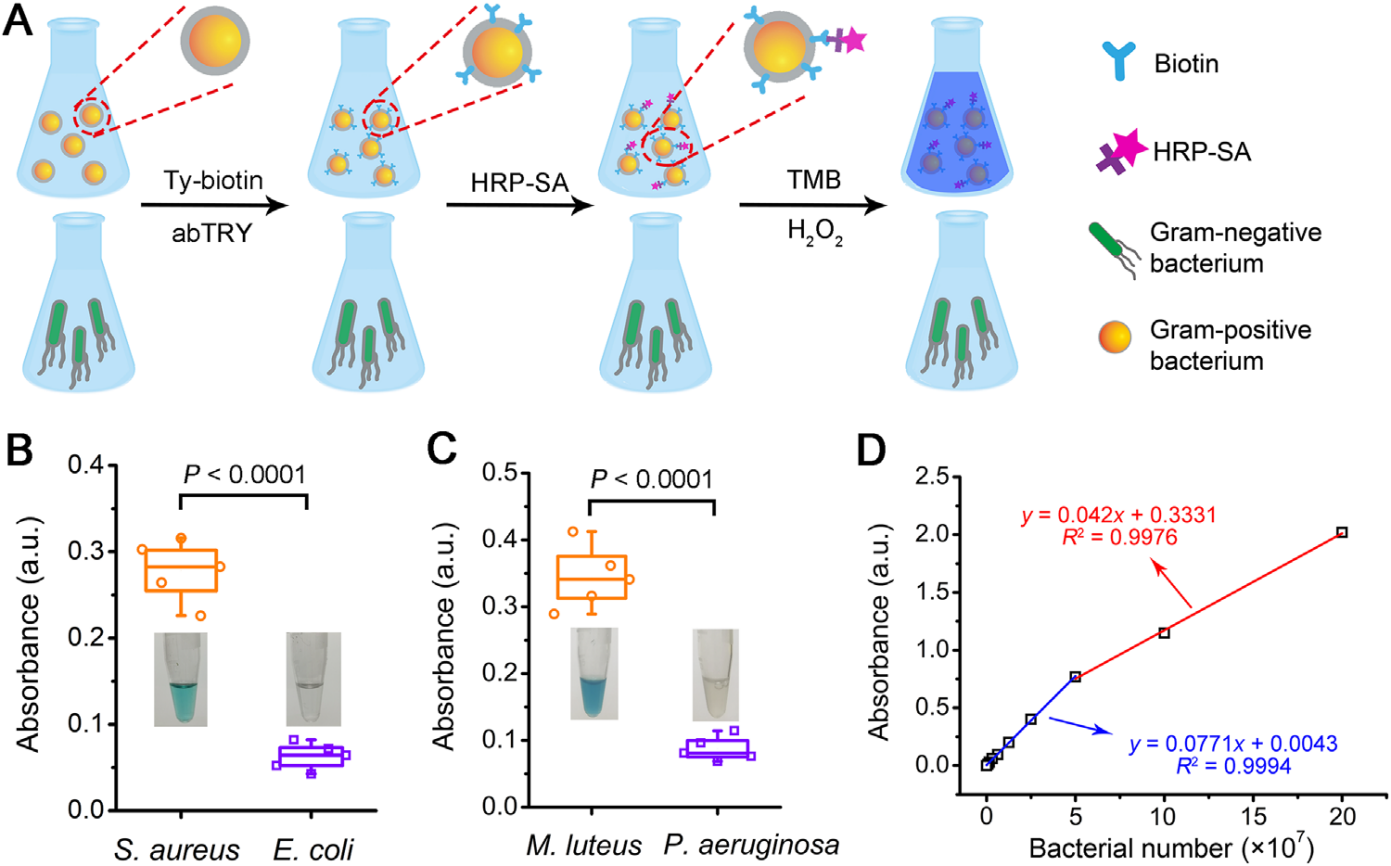
Naked‐eye detection of Gram‐positive bacteria. (A) Scheme illustrating the experimental procedures for naked‐eye differentiation of Gram‐positive bacteria from Gram‐negative bacteria. (B) Absorbance of the “TMB + H_2_O_2_” solutions after being added with *S. aureus* or *E. coli* bacteria that were first treated with Ty‐biotin (10 µg mL^−1^) and abTYR (0.17 µM) and then incubated with SA‐HRP (20 µg mL^−1^), followed by PBS washing for three times. Inset: representative photographs of the “TMB + H_2_O_2_” solutions added with *S*. *aureus* (left) or *E*. *coli* (right) bacteria receiving the abovementioned treatments. Statistical significance between the indicated groups was calculated using two‐sided unpaired *t*‐test. (C) Absorbance of the “TMB + H_2_O_2_” solutions after being added with *M. luteus* or *P. aeruginosa* bacteria subjected to the treatments described in (B). Inset: representative photographs of the “TMB + H_2_O_2_” solutions added with *M*. *luteus* (left) or *P*. *aeruginosa* (right) bacteria treated as mentioned above. Statistical significance between the indicated groups was calculated using two‐sided unpaired *t*‐test. (D) Plot of the absorbance of “TMB + H_2_O_2_” solutions versus the number of HRP‐labeled *S*. *aureus* bacteria that were added to the sensing system

Gram staining has been a basic and gold standard method for bacterial differentiation and classification in microbial biology. However, this traditional technique requires cumbersome experimental procedures and microscopic observation, which, if not operated skillfully, may lead to false‐positive/negative results.^[^ ^22^ ^]^ Therefore, developing alternative sensing methods to detect Gram‐positive and Gram‐negative bacteria is of great practical value. For this purpose, we sought to combine the TyOCR‐based modification method with horseradish peroxidase (HRP) to form a naked‐eye biosensing system (Figure 5A). This design rests on the excellent specificity of TyOCR‐based biotinylation toward Gram‐positive bacteria, allowing the facile labeling of SA‐conjugated HRP (SA‐HRP), and thus the resultant HRP‐modified Gram‐positive bacteria are poised to produce colorimetric signals in the presence of H_2_O_2_ and chromogenic substrates such as 3,3′,5,5′‐tetramethylbenzidine (TMB). Specifically, two Gram‐positive bacteria (*S*. *aureus* and *M*. *luteus*) and two Gram‐negative bacteria (*E*. *coli* and *P*. *aeruginosa*) were treated with Ty‐biotin and abTYR, followed by SA‐HRP modification. Photographs in Figure 5B,C show that the addition of the treated Gram‐positive bacterial suspensions into “TMB + H_2_O_2_” solutions efficiently triggered a change in color from colorless to blue, whereas the suspensions of the two Gram‐negative groups were still colorless. Quantitative data also confirmed the significant differences in absorbance between the samples of the Gram‐positive groups and those of the Gram‐negative groups (Figure 5B,C). Additionally, the absorbance of oxidized TMB solutions exhibited a highly linear correlation with the amount of the HRP‐modified Gram‐positive bacteria (Figure 5D and Figure S7). The limit of detection (LOD) values for *S*. *aureus* and *M*. *luteus* were calculated to be 1.14 × 10^6^ and 1.03 × 10^6^, respectively. Overall, compared with the traditional Gram staining method, this TyOCR‐based sensing system is eligible to serve as a promising alternative because of its facile operation, easy observation by naked eyes, and good reliability.

## 3 CONCLUSIONS

In summary, a cell surface modification strategy intended for Gram‐positive bacteria was rationally designed based on TyOCR. This strategy works by converting phenol‐tagged chemical reporters (i.e., fluorophores and biotin) into *o*‐quinone‐tagged forms in the presence of abTYR and molecular oxygen, and then linking the chemical reporters to TAs in Gram‐positive bacterial cell walls via *o*‐quinone‒amine chemical conjugation. Compared with the conventional NHS ester‒amine coupling method, TyOCR enables higher labeling efficiency/density for bacterial surface modification and sidestep the lability of NHS esters in aqueous solutions. We proved the versatility of the modification technique from multiple aspects, including fluorescence imaging, photodynamic inactivation, magnetic cell isolation, and naked‐eye bacterial differentiation. We believe that the as‐designed strategy expands the toolbox for bacterial cell surface modification and will find broad applications in the biomedical field and beyond.

## 4 EXPERIMENTAL SECTION

Experimental details are provided in the Supporting Information.

## CONFLICT OF INTEREST

The authors declare no conflict of interest.

## Supporting information

Supporting Information

## Data Availability

The data that support the findings of this study are available from the corresponding author upon reasonable request.
