# Supplementary material for: Direct chemical editing of Gram‐positive bacterial cell walls via an enzyme‐catalyzed oxidative coupling reaction
Source: Exploration (Beijing). 2022 May 28;2(5):20220010. doi: 10.1002/EXP.20220010 (PMC10190971; doi:10.1002/EXP.20220010)
Supplement: Supplementary file 1 — Supporting Information [file EXP2-2-20220010-s001.docx]

**Supporting Information**

**Direct chemical editing of Gram-positive bacterial cell walls via an enzyme-catalyzed oxidative coupling reaction**

Hao-Ran Jia, Ya-Xuan Zhu, Yi Liu, Yuxin Guo, Sayed Mir Sayed, Xiao-Yu Zhu, Xiaotong Cheng, and Fu-Gen Wu*

**Materials and strains.** Ty-Cy5, Ty-Cy3, and Ty-AF488 were purchased from AAT Bioquest (CA, USA). Ty-biotin, SDS, urea, LTA (purified from *S*. *aureus*), Tris-HCl were ordered from Sigma-Aldrich. Tyrosinase (abTYR), α-amylase, and pronase were bought from Shanghai Yuanye Bio-Technology Co., Ltd. (Shanghai, China). WGA-AF488 was ordered from Thermo Fisher Scientific Co., Ltd. (Invitrogen, Shanghai, China). Immidazole was bought from Biosharp (Anhui, China). DNase I was purchased from Beijing Biotopped Co., Ltd. (Beijing, China). Tunicamycin, *N*-hydroxysuccinimide (NHS), and NHS-biotin were purchased from Aladdin Reagent, Co., Ltd. (Shanghai, China). Compound 1771 was ordered from Enamine Ltd. (Product ID: T5526252). Ce6, 1-ethyl-3-(3-(dimethylamino)propyl)carbodimide (EDC), and TMB were ordered from J&K Scientific Ltd. NHS-Cy5 was purchased from Nanjing Bioorth Biotech Co., Ltd. (Nanjing, China). Avidin was bought from Sangon Biotech (Shanghai, China). SA-SIO NPs with an average diameter of 50 nm were purchased from Biotyscience Co., Ltd. (Beijing, China). Avidin-FITC and SA-HRP were bought from Beijing Biosynthesis Biotechnology Co., Ltd. (Beijing, China). *S*. *aureus*, *M*. *luteus*, *B*. *subtilis*, *E*. *coli*, and *P*. *aeruginosa* were originated from China General Microbiological Culture Collection Center (CGMCC). *P*. *vulgaris* was ordered from Guangdong Microbial Culture Collection Center (GDMCC). All bacteria were cultured in LB media under shaking at 37℃ except *M*. *luteus* that was grown at 30℃.

**Tyrosinase-mediated fluorescence labeling of bacteria.**

Before experiments, tyrosinase stock solutions were first prepared by dissolving tyrosinase powder in PBS at 2 mg mL^−1^ and stored at −80 ℃. Bacteria grown in fresh LB media were collected when they reached the exponential growth phase. The bacteria were then centrifuged at 6000 rpm for 5 min and washed by PBS twice. Next, for each type of bacterial strains, the bacterial suspension was mixed with an equal volume of Ty-Cy5 solution to reach a final Ty-Cy5 concentration of 1 μg mL^−1^. After vigorous vortex, the mixture was further treated with abTYR at a concentration of 0.17 μM at room temperature for 10 min. Before fluorescence imaging, the treated bacteria were washed by PBS for 3 times to remove tyrosinase as well as unreacted dyes. Likewise, Ty-Cy3 and Ty-AF 488 were used to label *S*. *aureus* bacteria based on the same staining procedure. The treated bacteria were observed under an inverted confocal laser scanning microscope (TCS SP8, Leica, Germany), and their fluorescence intensities were quantified using a flow cytometer (NovoCyte 2070R, ACEA Bioscience, Inc., USA). To test the specificity of TyOCR staining, a mixed suspension of *E*. *coli* and *S*. *aureus* bacteria was first washed by PBS twice, and then incubated with Ty-Cy5 (1 μg mL^−1^) and abTYR (0.17 μM) at room temperature for 10 min. The treated bacteria were washed by PBS for 3 times, followed by flow cytometric analysis and confocal imaging.

To rule out the involvement of physical interactions between the fluorophore and Gram-positive bacterial surface, we carried out the following experiment. First, the above-prepared Cy5-labeled *S*. *aureus* bacteria were suspended in PBS (control), 0.1% SDS, 0.5 M NaCl, and 50 mM urea solutions, respectively. After incubation for 20 min at room temperature, the treated bacteria were centrifuged and resuspended in PBS, followed by flow cytometric analysis. We then confirmed the chemical reaction mechanism of this labeling strategy as follows. *S*. *aureus* bacteria were treated with Ty-Cy5 (1 μg mL^−1^) and abTYR (0.17 μM) in the presence or absence of aniline (5 mM) at room temperature for 10 min. Afterward, the bacteria were washed by PBS for 3 times, followed by confocal imaging and flow cytometric analysis. For the same purpose, we first prepared hypoxic and deoxygenated PBS solutions using zero oxygen tablets (Mettler Toledo, Switzerland) according to the manufacture’s protocol. Then, *S*. *aureus* bacteria were suspended in hypoxic and deoxygenated PBS solutions, respectively, followed by the addition of Ty-Cy5 and abTYR as mentioned above. After incubation for 10 min, the treated bacteria were collected and characterized by confocal microscopy and flow cytometry.

**Preparation of fluorescence-labeled bacterial sacculi.** Briefly, different Gram-positive bacteria (*S*. *aureus*, *M*. *luteus*, and *B*. *subtilis*) were first labeled by Ty-Cy5 via the abTYR-mediated coupling reaction as described above, and then subjected to sacculus preparation based on a previously published method.^1^ For example, Cy5-labeled *S*. *aureus* bacterial suspension was added into a boiling SDS solution (5% w/v) drop by drop with stirring for 30 min. Next, the mixture was centrifuged at 8000 rpm for 10 min and the insoluble material was resuspended in a 4% w/v SDS solution, which was boiled again with stirring for another 30 min. After being washed by water for 3 times, the sample was further treated with a mixed solution containing 10 mM Tris-HCl, 10 mM NaCl, 100 μg mL^−1^ α-amylase, 0.32 M immidazole, 50 μg mL^−1^ DNase I, and 1 mM MgSO_4_ at 37℃ for 2 h. Then, the resultant dispersion was centrifuged again and resuspended in a 0.05 M Tris-HCl solution (pH 7.8) containing 1.4 mg mL^−1^ pronase, followed by incubation at 60℃ for 2 h. Finally, the sample was again collected and treated with a boiling SDS solution (1% w/v) for 30 min. For confocal imaging, the purified bacterial sacculi should be further stained by WGA-AF488 (5 μg mL^−1^) for 15 min and washed by water for 3 times.

**HF treatment of bacteria.** To begin with, Cy5-labeled *S*. *aureus* bacterial sacculi, which were prepared as described above, were suspended in 48% HF to remove their TA. After incubation at 4℃ for 48 h, the sample was washed by water for 3 times. For comparison, Cy5-labeled sacculi were suspended in water and then subjected to the same treatments. Before imaging, the sacculi were stained by WGA-AF488 (5 μg mL^−1^) to indicate cell wall structures. The Cy5 fluorescence signals of these samples were recorded by confocal microscopy and flow cytometry.

**Inhibition of TA biosynthesis in *S***. ***aureus*.** To inhibit the biosynthesis of WTA in *S*. *aureus*, tunicamycin was added into fresh LB media at a predetermined concentration (0, 0.01, 0.04, or 0.08 μg mL^−1^) for bacterial culture. After 8 h of incubation, the bacteria were pelleted and washed with PBS for 3 times, followed by the TyOCR-based labeling of Ty-Cy5 as described above. The fluorescence intensities of the bacteria were measured by flow cytometry. For confocal fluorescence imaging, the Cy5-labeled bacteria were further stained by WGA-AF488 (5 μg mL^−1^) to label cell walls, and then washed by PBS for 3 times. To inhibit the biosynthesis of LTA in *S*. *aureus*, compound 1771 was added into fresh LB media at a predetermined concentration (0, 0.5, 1, 5, and 10 μg mL^−1^) for bacterial culture. After incubation for 8 h, the treated bacteria were washed by PBS for 3 times and labeled by Ty-Cy5 as described above. The fluorescence signals of these samples were quantified by flow cytometry.

**Synthesis and characterization of Cy5-LTA.** Initially, 0.5 mg Ty-Cy5 was dissolved in 2 mL PBS (pH 7.4) and mixed with 50 μL abTYR solution (2 mg mL^−1^). After incubation under shaking at room temperature for 15 min, the mixture was subjected to ultrafiltration with a molecular weight cut-off (MWCO) of 10 kDa at 4000 rpm for 10 min to remove abTYR. The resultant *o*-quinone-Cy5 (oxidized Ty-Cy5) solution was collected and reacted with 5 mg LTA (dissolved in 1 mL of water) at room temperature for 30 min. Next, the solution was dialyzed (MWCO: 2 kDa) against water at 4℃ for 3 days to remove unreacted dyes, followed by lyophilization. The obtained product was characterized by UV–vis spectroscopy and ^1^H NMR spectroscopy using a UV–vis spectrophotometer (UV-2600, Shimadzu, Japan) and a Bruker Avance 500 MHz instrument, respectively.

**Ethanol/EDTA treatment of Gram-negative bacteria.** First, *E*. *coli* and *P*. *aeruginosa* bacteria grown to the exponential growth phase were collected and washed by PBS for 3 times. Then, these bacteria were treated with 10 mM EDTA for 30 min or 95% ethanol for about 30 s, followed by centrifugation. The collected bacteria were next incubated with Ty-Cy5 and abTYR as described above, and washed by PBS for 3 times. Confocal imaging was carried out to observe their fluorescence signals.

**Tyrosinase-mediated biotinylation of bacteria.** Bacteria grown in fresh LB media were collected in the exponential growth phase and washed by PBS for 3 times. Next, for each type of bacterial strains, the bacterial suspension was mixed with an equal volume of Ty-biotin solution to reach a final Ty-biotin concentration of 10 μg mL^−1^. After vigorous vortex, the mixture was further treated with abTYR (0.17 μM) at room temperature for 10 min, followed by PBS washing for 3 times. To evaluate the potential toxic effect of the biotinylation to bacterial cells, *S*. *aureus* bacteria were labeled by different concentrations of Ty-biotin as described above. The cell viabilities were assessed by the LB agar plating assay according to standard procedures.

**Comparison of the NHS ester‒amine reaction and TyOCR in bacterial surface modification.** For the bacterial labeling of NHS-Cy5, different bacterial suspensions were separately mixed with an NHS-Cy5 solution to reach a final NHS-Cy5 concentration of 2 μM. After incubation at room temperature for 20 min, the bacteria were washed with PBS for three times and subjected to confocal fluorescence imaging. To compare the labeling efficiencies of the two reactions, we treated bacteria with determined concentrations of NHS-Cy5 or Ty-Cy5 (in the presence of 0.17 μM abTYR). Then, the fluorescence intensities of the treated bacteria were measured by flow cytometry at determined reaction time points. For the same purpose, *S*. *aureus* bacteria were incubated with different concentrations of NHS-biotin or Ty-biotin (in the presence of 0.17 μM abTYR) for 20 min. Next, the treated bacteria were washed by PBS for three times, and further incubated with 5 μg mL^−1^ avidin-FITC for 10 min, followed by flow cytometric analysis. To evaluate the stability of NHS-Cy5 and Ty-Cy5 in aqueous solutions, the two probes were freshly prepared as stock solutions (100 μM in PBS, pH = 7.4) and kept at room temperature. Next, the stock solutions of the two probes were withdrawn at different time points, and *S*. *aureus* bacteria were stained as described above. The fluorescence intensities of the treated bacteria were measured by flow cytometry.

**Synthesis of avidin-Ce6.** First, to activate the carboxyl groups of Ce6, 3.5 mg Ce6 dissolved in 0.5 mL DMSO was mixed with 6.1 mg EDC (in 0.5 mL DMSO) and 13.2 mg NHS (in 0.5 mL DMSO). After reaction for 2 h, 107 μL of the above mixture was withdrawn and added into 4 mL of avidin solution (1.25 mg mL^−1^ in PBS, pH 7.4) to react at room temperature for 12 h. Next, the solution was dialyzed (MWCO: 10 kDa) against water at 4℃ for 3 days and freeze-dried for further use.

**Photodynamic inactivation of Gram-positive bacteria.**

To begin with, *S*. *aureus* bacteria were treated with Ty-biotin (10 μg mL^−1^) and abTYR (0.17 μM) for 10 min, and washed by PBS for 3 times. Then, the biotinylated bacteria were incubated with avidin-Ce6 (Ce6: 5 μg mL^−1^) for 10 min, and further washed by PBS for 3 times. These Ce6-labeled bacteria were divided into several groups, and each group received white light irradiation (5 mW cm^−2^) for a predetermined time period (0, 0.5, 1, 3, 5, or 10 min). The survival rates of the bacteria were quantified via the LB agar plating assay.

For the selective killing of *S*. *aureus* bacteria in the presence of *E*. *coli* bacteria, mixed suspensions of the two bacteria were first treated with different concentrations of Ty-biotin and abTYR (0.17 μM) for 10 min, and then washed by PBS for 3 times. Next, the bacteria were incubated with avidin-Ce6 (Ce6: 5 μg mL^−1^) for 10 min, washed by PBS for 3 times, and subsequently irradiated by white light (5 mW cm^−2^) for 3 min. Bacteria receiving the above treatments except light irradiation were set as the control group. For each group, the proportions of *S*. *aureus* and *E*. *coli* were measured by counting the CFUs grown on LB and EMB agar plates.

**Magnetic separation of Gram-positive bacteria.** To begin with, *S*. *aureus* and *E*. *coli* bacteria were separately stained by Hoechst 33342 (10 μg mL^−1^) and SYTO 9 (5 μM) for 15 min, followed by PBS washing. The two bacterial suspensions were mixed (1:1, v/v) and then treated with 10 μg/mL Ty-biotin and 0.17 μM abTYR at room temperature for 10 min. After PBS washing for 3 times, 0.5 mL of the bacterial suspension (~1 × 10^7^ cells) was mixed with an equal volume of 0.1 mg mL^−1^ SA-SIO NPs, and the resultant mixture was incubated at room temperature for 15 min. For magnetic separation, the sample was added into an MACS MS column placed on a MiniMACS separator (Miltenyi Biotec, Germany), and the bacterial suspension dropped to the bottom tube was collected. Then, the collected suspension was transferred into a quartz cuvette, placed under a UV lamp, and imaged using a camera. In addition, the Hoechst 33342-stained *S*. *aureus* suspension, SYTO 9-stained *E*. *coli* suspension, and their mixture were also imaged accordingly. For fluorescence spectroscopy, the bacterial mixture was first biotinylated as described above, and then incubated with SA-SIO NPs at a predetermined concentration (0, 10, 25, 50, or 75 μg mL^−1^). After magnetic separation, the bacterial suspension collected in the bottom tube was characterized by fluorescence spectroscopy using an RF-5301PC spectrofluorophotometer (Shimadzu, Japan).

To improve the bacterial isolation efficiency of this method, we carried out 3 rounds of magnetic separation. To be specific, the SA-SIO NP-labeled bacterial sample was added into a separation column placed on a MiniMACS separator to cross the column for magnetic isolation. Next, the column was taken out from the separator and added with fresh PBS to rinse those isolated bacteria from the column. The collected bacterial suspension was vigorously pipetted and added to a new separation column to receive a second round of separation as described above. These steps were repeated until 3 rounds of separation were finished. The isolated bacteria after each round of magnetic separation were cultured on LB and EMB agar plates, and their CFUs were counted to evaluate the separation efficiency.

To evaluate the cell viabilities of bacteria after magnetic separation, *S*. *aureus* was first labeled with SA-SIO NPs as described above and then subjected to 1, 2, or 3 rounds of magnetic separation. Unlabeled bacteria without receiving magnetic separation were set as the control. Next, the number of bacterial cells in each group was quantified by flow cytometry, and their viabilities were evaluated through the spread plate method with the same starting number of bacteria.

**SEM and ICP-MS measurements.** Typically, *S*. *aureus* bacteria were separately conjugated with different concentrations of Ty-biotin (0, 0.5, 5, and 10 μg mL^−1^) via the abTYR-mediated coupling reaction as described above. After PBS washing for 3 times, the biotinylated bacteria were incubated with SA-SIO NPs at a concentration of 50 μg mL^−1^ for 15 min. To remove unbound SA-SIO NPs, the bacteria were pelleted and again washed by PBS for 3 times. Next, the samples were fixed in 2.5% glutaraldehyde solutions overnight and gradually dehydrated using graded ethanol solutions according to standard treatment procedures. These SA-SIO NP-modified *S*. *aureus* bacteria were then observed by SEM using a Zeiss Ultra Plus scanning electron microscope (Carl Zeiss, Germany). For quantitative analysis, *S*. *aureus* bacteria were first separately labeled by different concentrations of Ty-biotin (0, 0.5, 2, 5, and 10 μg mL^−1^) and then modified by SA-SIO NPs as described above. The Fe content in each sample was measured via ICP-MS using an inductively coupled plasma mass spectrometer (ICPMS 7700, Agilent). Before measurement, flow cytometry was used to record the number of bacteria in these samples.

**Naked-eye detection of Gram-positive bacteria.** First, bacteria grown in fresh LB media were collected in the exponential growth phase and washed by PBS for 3 times. Next, the bacteria were treated with Ty-biotin (10 μg mL^−1^) and abTYR (0.17 μM) at room temperature for 10 min. After being washed by PBS for 3 times, the treated bacteria were further incubated with SA-HRP (20 μg mL^−1^) for 5 min and again subjected to 3 times of PBS washing to remove unbound SA-HRP. Before sensing, the substrate solution containing 250 μL TMB solution (2 mg mL^−1^ in ethanol), 5 mL buffer (0.2 M Na_2_HPO_4_, 0.1 M citrate, pH = 5.0), and 50 μL 0.75% H_2_O_2_ solution was freshly prepared. Afterward, different quantities of the bacteria were separately added into 300 μL of the substrate solution, and the resultant mixtures were incubated at room temperature for 3 min, followed by image acquisition by a camera. For absorbance measurement, 10 μL of 2 M H_2_SO_4_ solution was added to each sample and the absorbance at 450 nm was recorded using a microplate reader (Multiskan FC, Thermo Scientific, USA).

**Statistical analysis.** All the statistical data were presented as the mean ± standard deviation (SD) from at least three independent experiments. The significance between two groups was analyzed by two-tailed Student’s *t*-test. For multiple comparisons, one-way analysis of variance (ANOVA) with Tukey’s post-hoc test was adopted. *P* values of less than 0.05 were considered as significant difference.


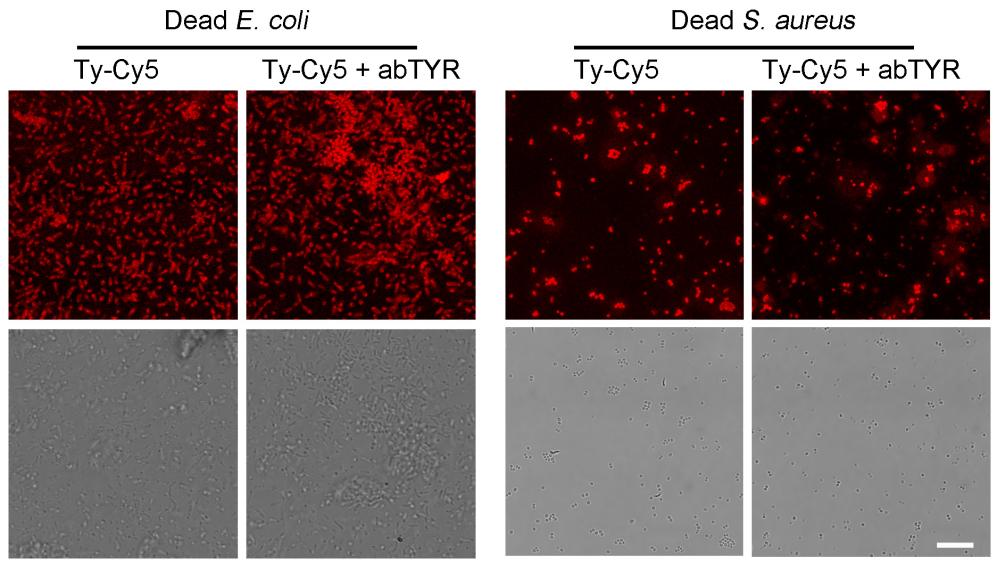


**Figure S1.** Confocal images of dead *E*. *coli* and *S*. *aureus* bacteria after treated with either Ty-Cy5 (1 μg mL^−1^) alone or Ty-Cy5 (1 μg mL^−1^) plus abTYR (0.17 μM) at room temperature for 10 min. Scale bar = 25 μm.


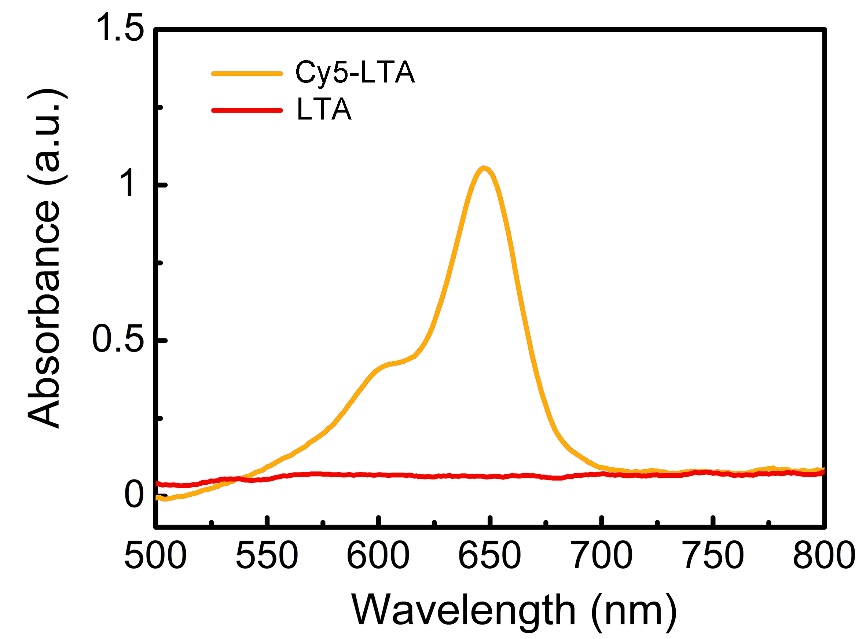


**Figure S2.** UV‒vis spectra of LTA (0.5 mg mL^−1^ in water) and Cy5-LTA (0.5 mg mL^−1^ in water).


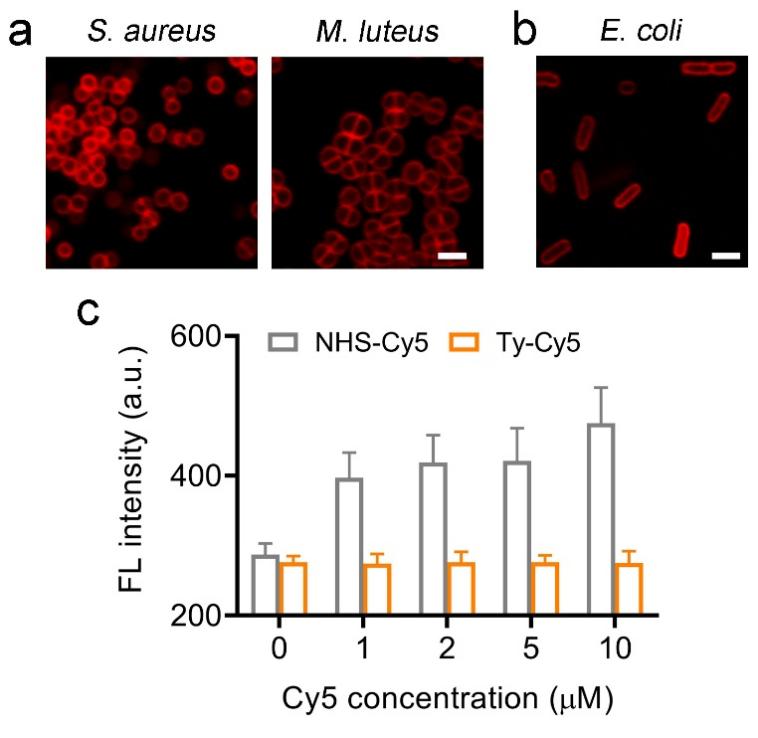


**Figure S3.** (a) Confocal fluorescence images of two Gram-positive bacteria (*S*. *aureus* and *M*. *luteus*) after being stained by NHS-Cy5 (2 μM) for 20 min. Before imaging, the stained bacteria were washed with PBS for three times. Scale bar = 2 μm. (b) Confocal fluorescence image of *E*. *coli* bacteria after the same treatment as indicated above. Scale bar = 2 μm. (c) Fluorescence intensities of *E*. *coli* bacteria that were treated with various concentrations of NHS-Cy5 or Ty-Cy5 (with the addition of 0.17 μM abTYR) for 20 min, as measured by flow cytometry.


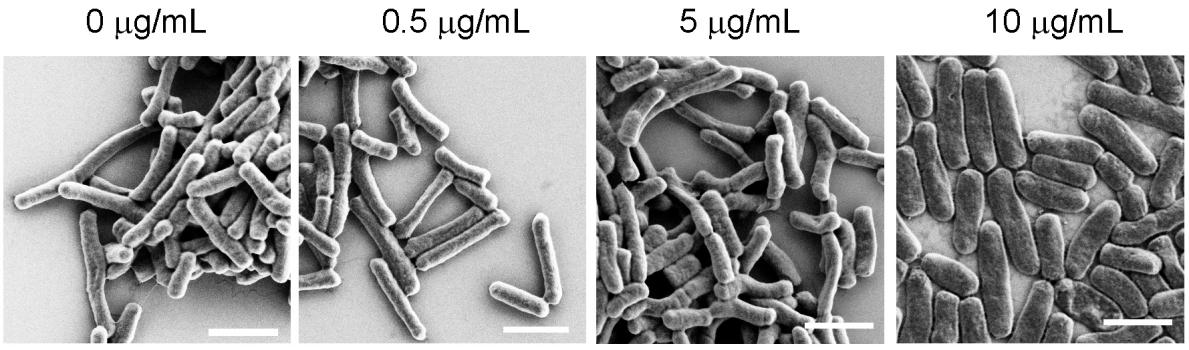


**Figure S4.** SEM images of *E*. *coli* bacteria that were first treated with the indicated concentrations of Ty-biotin and abTYR (0.17 μM) and then incubated with SA-SIO NPs (50 μg mL^−1^). Scale bars = 2 μm.


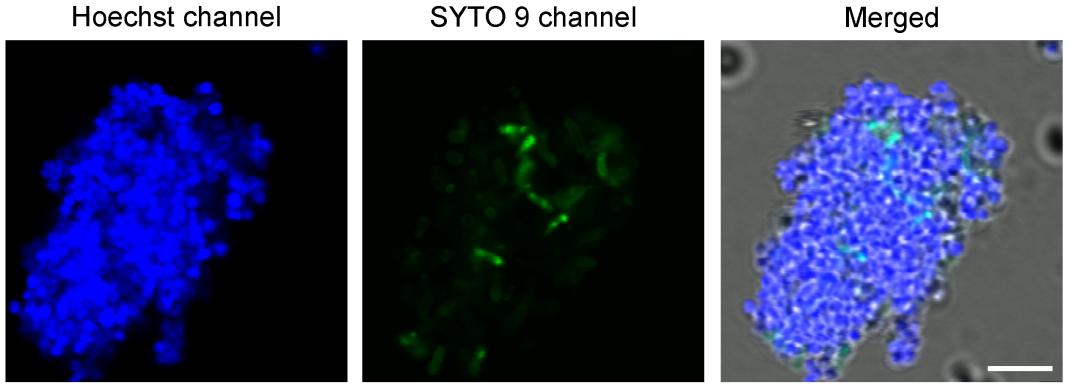


**Figure S5.** Confocal images showing the presence of a few *E*. *coli* bacteria (labeled by SYTO 9) in an aggregate of *S*. *aureus* bacteria (labeled by Hoechst 33342) after 1 round of magnetic separation. Scale bar = 5 μm.

**
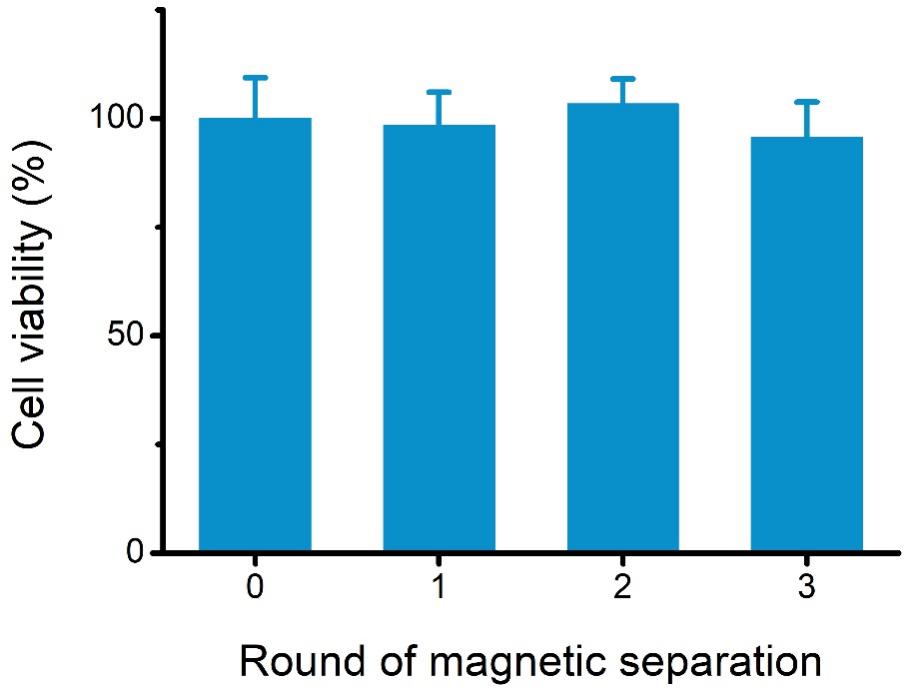
**

**Figure S6.** Cell viabilities of SA-SIO NP-modified *S*. *aureus* bacteria after 1, 2, or 3 rounds of magnetic separation. Untreated bacteria with 0 round of magnetic separation were set as the control group.

**
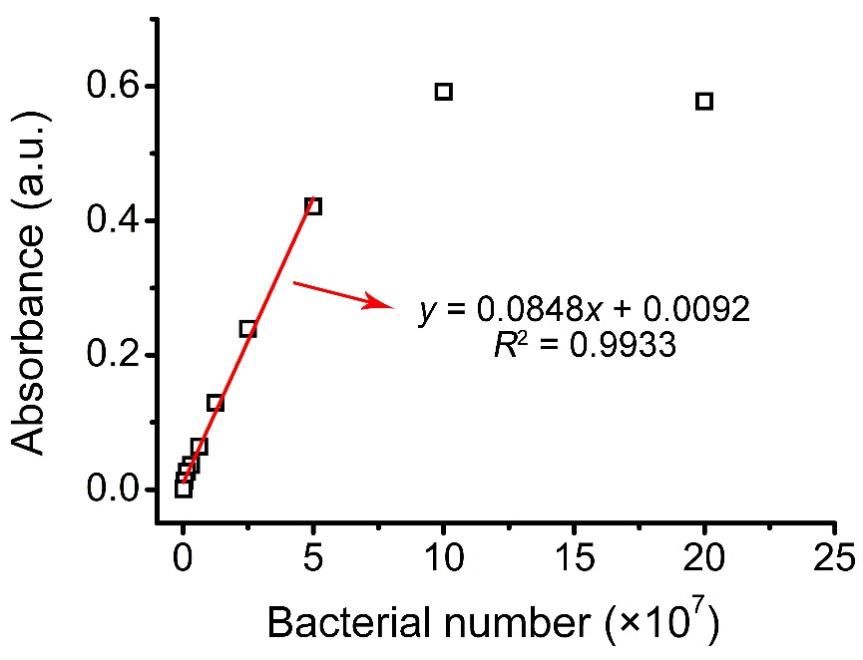
**

**Figure S7.** Plot of the absorbance of “TMB + H_2_O_2_” solutions versus the number of HRP-labeled *M*. *luteus* bacteria.

**Reference**

1. E. Kuru, H. V. Hughes, P. J. Brown, E. Hall, S. Tekkam, F. Cava, M. A. de Pedro, Y. V. Brun, M. S. VanNieuwenhze, *Angew*. *Chem*. *Int*. *Ed*. **2012**, ***51***, 12519–12523.
